# Remediation of Rare Earth Element Pollutants by Sorption Process Using Organic Natural Sorbents

**DOI:** 10.3390/ijerph120911278

**Published:** 2015-09-10

**Authors:** Monica Butnariu, Petru Negrea, Lavinia Lupa, Mihaela Ciopec, Adina Negrea, Marius Pentea, Ionut Sarac, Ionel Samfira

**Affiliations:** 1 Banat’s University of Agricultural Sciences and Veterinary Medicine “King Michael I of Romania” from Timisoara, Calea Aradului 300645, Romania; E-Mails: pentea1967@yahoo.com (M.P.); ionut_sarac@yahoo.com (I.S.); samfiraionel@yahoo.ro (I.S.); 2 Politehnica University Timisoara, Faculty of Industrial Chemistry and Environmental Engineering, P–Ta Victoriei 2, Timisoara 300006, Romania; E-Mails: negrea_petru@yahoo.ca (P.N); lavinia.lupa@upt.ro (L.L.); mihaela.ciopec@upt.ro (M.C.); adina_n_19@yahoo.co.uk (A.N.)

**Keywords:** rare earth element, bone powder, model isotherms

## Abstract

The effects of the sorption of environmental applications by various source materials of natural organic matter, *i.e.*, bone powder, was examined. Sorption capacities and subsequent rare earth element retention characteristics of all metals tested were markedly increased by ionic task-specific. In this study, the abilities of three models’ isotherms widely were used for the equilibrium sorption data: Langmuir, Freundlich and Redlich-Peterson. For all studied metal ions the maximum adsorption capacity is close to those experimentally determined. The characteristic parameters for each isotherm and related coefficients of determination have been determined. The experimental data achieved excellent fits within the following isotherms in the order: Langmuir > Redlich-Peterson > Freundlich, based on their coefficient of determination values. The bone powder has developed higher adsorption performance in the removal process of Nd(III), Eu(III), La(III) from aqueous solutions than in the case of the removal process of Cs(I), Sr(II) and Tl(I) from aqueous solutions. The described relationships provide direct experimental evidence that the sorption-desorption properties of bone powder are closely related to their degree of the type of the metal. The results suggest a potential for obtaining efficient and cost-effective engineered natural organic sorbents for environmental applications.

## 1. Introduction

Globally there is a tendency to develop simple, fast, cheap and effective natural organic matter (NOM) methods for ensuring their application *in-situ* absorption for rare earth element (REE) or radionuclides. Sorption materials are used as materials to prevent propagation and dispersion REE [1].

In accordance with American Society for Testing and Materials (ASTM) and The United States Environmental Protection Agency (US EPA), sorption materials like bone powder are natural insoluble materials and mixtures of materials used for recovering mechanisms of absorption, adsorption or both [2]. Studies have shown the superiority of natural sorbents towards the synthetic organic in their application for retaining of REE (*i.e.*, Nd(III), Cs(I), Sr(II), Tl(I), Eu(III), La(III)), given their capacity to biodegradation [3]. Organic natural sorbents have the following characteristics: is biodegradable, renewable, low cost, low environmental impact, and readily manipulated [4]. A biodegradable product shows ecological, scientific and economic importance [5]. Organic natural sorbents science as a branch of interdisciplinary field of physical chemistry has had explosive growth, on the theoretical and actual application of modern technologies [6]. Because of the background depletion of reserves on the planet, current research is aimed at using renewable raw materials and the production of biodegradable materials (*i.e.*, bone powder) [7]. From this perspective, animal raw materials and especially organic natural sorbents have very interesting properties such as biodegradability [8], biocompatibility [9], selective permeability and physical–mechanical properties change [10]. These properties allow their application in different areas of economic interest in all sectors of modern industry [11]. Natural sorbents selection is complicated by overlapping requirements chemical, morphological, biological and surface structure requiring deep knowledge of relations-properties-biological effects and an integrated interdisciplinary study–chemical, physical, biological and clinical [12].

Considering the current issues related to environmental protection, the aim of this work is oriented in the direction of natural sorbents prepared from bone meal and REE adsorption characterization modern methods involving the application of fractal theory to analyze inductively coupled plasma mass spectrometry.

## 2. Experimental Section

The bone powder (manufactured in the lab) was used as adsorbent material in the removal process of various metal ions: Nd(III), Cs(I), Sr(II), Tl(I), Eu(III), La(III) from aqueous solutions. The adsorption process of metal ions onto the bone powder has been performed by the static, so called “batch” mode, using a Julabo SW23, GmbH, 77960 Seelbach / Germany, mechanical shaker bath. The quantity of 0.5 g of the bone powder was equilibrated for 1 hour with 25 mL of metal ions solutions having various concentrations (5–500 mg/L for Nd(III), Eu(III), La(III); 5–400 mg/L for Tl(I) and 5–200 mg/L for Cs(I), Sr(II) ions). The experiments were performed at room temperature 25 °C and the quantity of metal ions remaining in the liquid phase after equilibration was measured by inductively coupled plasma mass spectrometry (ICP–MS Bruker Aurora M90, Bruker Corporation, Germany). The difference between the initial and equilibrium mass concentrations of metal ions was used for the calculation of the adsorbed amount of metal ions on 1 g of bone powder, taking into account the mass of the bone powder, volume and mass concentration of the solution. The adsorbed amount of metal ions onto bone powder was determined using the following Equation (1):

(1) $$q_{e}=\frac{\left(C_{0}-C_{e}\right)V}{m}$$

where: *q_e_* is the adsorbed amount of metal ions onto bone powder (mg/g), *C_0_* and *C_e_* is the initial and the equilibrium concentration of metal ions in the solution (mg/L), *V* is the volume of the solution (L), and m is the weight mass of the bone powder (g). In order to provide some insight information regarding the sorption mechanism, the surface properties and affinity of the bone powder for the studied metal ions three equilibrium isotherm equations were used to describe the experimental sorption data. Thus, an accurate mathematical description of the equilibrium isotherm, preferably based on a correct sorption mechanism, is essential to the effective design of sorption systems.

For isotherms adsorption interpretation there are several empirical models. In this study, the abilities of three widely used isotherms: Langmuir, Freundlich and Redlich–Peterson to model the equilibrium sorption data were examined. The Langmuir model is a model based on the fact that on the surface of the sorbent is formed monomolecular layer of adsorbate, and all active sites are of equal energy and enthalpy of adsorption [13]. The Langmuir equation is as follows (2):

(2) $$q_{e}=\frac{q_{m}K_{L}C_{e}}{1+K_{L}C_{e}}$$

where *K_L_* is the sorption equilibrium constant (L/mg).

The Freundlich expression [14] is an empirical equation applicable to non–ideal sorption on heterogeneous surface as well as multilayer sorption, and is expressed by the following Equation (3):

(3) qe=KFCe1n

where *K_F_* ((mg/g)(L/mg)^1/n^)) and *1/n* are the Freundlich constants related to the sorption capacity and sorption intensity, respectively. The Freundlich parameter *1/n*, should have values <1 for classification as favorable adsorption. The Redlich–Peterson model [15] combines models of Freundlich and Langmuir and intends to describe in addition of the heterogeneity of the sorbent surface and a certain number of adsorption sites with the same adsorption potential [16].

The Equation (4) Redlich–Peterson is the following:

(4) $$q_{e}=\frac{K_{R}C_{e}}{1+\alpha C_{e}^{\beta }}$$

where *K_R_* (L/g) and α (L/mol) are the Redlich–Peterson isotherm constants, while *β* is the exponent, which lies between 0 and 1. When β = 1, Equation (4) is the same as the Langmuir isotherm equation, which assumed the monolayer coverage of adsorbate over a homogenous adsorbent surface (a basic assumption is that sorption takes place at specific homogeneous sites within the adsorbent).

Accordingly, the observed β value higher than 1, could be attributed to a heterogeneous predominated surface adsorption, whereas homogeneous predominated surface adsorption occurred when β < 1. Redlich–Peterson has three constants, and therefore it is impossible to use its linear form to determine the constants.

In this case, we used a procedure to maximize it. In this study, three non–linear error functions were examined and in each case a set of isotherm parameters were determined by minimizing the respective error function across the concentration range studied.

The error functions employed were as follows:

(a) The Sum of the Squares of the Errors (*SSE*):

(5) $$\sum_{i=1}^{p}{\left(q_{e-meas}-q_{e-calc}\right)}_{i}^{2}$$

(b) The Average Relative Error (*ARE*):

(6) $$\sum_{i=1}^{p}\left|\frac{q_{e-meas}-q_{e-calc}}{q_{e-calc}}\right|$$

(c) The Sum of the Absolute Errors (*EABS*):

(7) $$\sum_{i=1}^{p}\left|q_{e-meas}-q_{e-calc}\right|$$

## 3. Results and Discussion

In order to assess different isotherms and their ability to correlate with experimental results, the plots from each isotherm have been fitted with the experimental data for sorption of studied metals ions onto bone powder.

The characteristics of bone powder, manufactured in the lab were: the humidity from the bone powder according to data obtained after proximate analysis was relatively small (2.09 ± 0.02); complex lipids was 4.93 ± 0.01, the protein content of the bone powder was almost 14 % (13.94 ± 0.04); the quantity of calcium and phosphorous from the sample (values are expressed in wt. %) was 13.4 Ca % and 7.2 P %. The Ca/P atomic ratio is about 1.46, which is lower than expected for the hydroxyapatite phase (1.67) [17].

**Figure 1:**
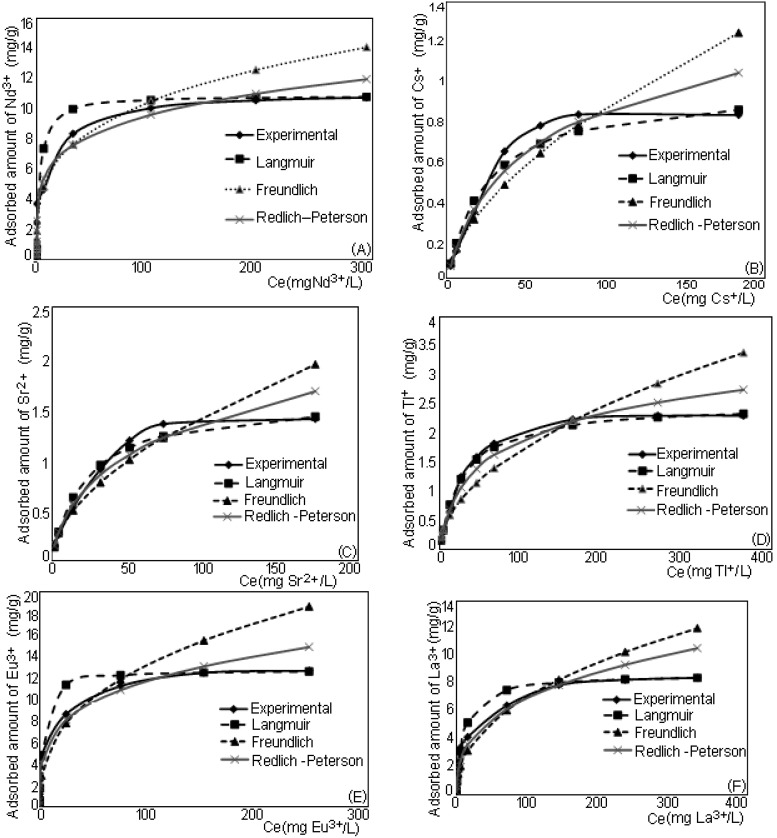
Experimental data and the sorption isotherm models for various metal ions adsorption onto bone powder (**A**) Nd (III), (**B**) Cs(I), (**C**) Sr(II), (**D**) Tl(I), (**E**) Eu(III), (**F**) La(III).

The characteristic of aqueous solution was the initial pH of solutions. Depending on the concentration of the initial solution, the pH ranged 1.5–3. At low concentrations of the solution the initial pH was 2.8–3, while at higher initial concentrations, pH was between 1.5–1.8. The pH was not adjusted and its influence on the adsorption capacity was not studied. The initial and final pH was read and after adsorption the values were the same. The experimental data of the amount of each metal ion adsorbed onto the bone powder were fitted to the isotherm models and the graphical representations of these models are presented in Figure 1, where *C_e_* is the equilibrium concentration of metal ions in the solution.

Comparing the correlation coefficient (*R^2^*) obtained for the studied isotherm for all adsorbed metal ions, it can be highlighted that the Langmuir model fits best for the description of metal ions sorption onto bone powder. This is followed by the Redlich–Peterson model, and the worst fit is obtained for the Freundlich isotherm model. All isotherm parameters obtained for the sorption of metal ions onto bone powder are presented in Table 1.

**Table 1:** Isotherm parameters obtained for the sorption of metal ions onto bone powder.

Isotherm	Parameter	Metal Ions					
		Nd(III)	Cs(I)	Sr(II)	Tl(I)	Eu(III)	La(III)
Langmuir	*q_m–meas_ (mg/g)*	10.75	0.84	1.39	2.29	12.6	8.43
	*q_m–calc_(mg/g)*	10.9	0.98	1.61	2.5	12.7	8.70
	*K_L_ (L/mg)*	0.349	0.0424	0.0444	0.0371	0.339	0.094
	*R^2^*	0.9980	0.9850	0.9920	0.9980	0.9970	0.9970
	*SSE*	25.7	0.0246	0.0312	0.033	16.3	2.66
	*ARE*	5.41	0.827	0.611	0.498	4.28	1.92
	*EABS*	12.2	0.369	0.391	0.458	9.15	3.59
Freundlich	*1/n*	0.276	0.571	0.548	0.515	0.374	0.434
	*K_F_ ((mg/g)(L/mg)^1/n^)*	2.965	0.0637	0.115	0.164	2.368	0.968
	*R^2^*	0.9100	0.9270	0.9570	0.9150	0.8550	0.938
	*SSE*	20.41	0.214	0.362	1.994	54.26	20.3
	*ARE*	5.41	1.326	1.052	2.193	3.10	2.586
	*EABS*	10.28	0.824	1.065	3.069	15.97	9.695
Redlich–Peterson	*β*	0.794	0.832	0.718	0.817	0.740	0.668
	*α (L/mol)*	83.93	0.075	0.2315	0.0938	107.23	2.337
	*K_R_ (L/g)*	314	0.039	0.100	0.102	380	3.63
	*R^2^*	0.9920	0.9730	0.9800	0.9790	0.995	0.984
	*SSE*	4.62	0.061	0.116	0.361	7.41	7.156
	*ARE*	1.81	0.662	0.652	0.849	1.639	1.41
	*EABS*	5.62	0.452	0.656	1.343	6.41	5.62

*q_m*-cal*_*—calculated adsorption capacity; *K_L_*—sorption equilibrium constant; *K_F_*—Freundlich constants; β—exponent; *K_R_*—Redlich–Peterson isotherm constants; *R^2^*—correlation coefficient; *SSE*—Sum of the Squares of the Errors; *ARE*—Average Relative Error; *EABS*—Sum of the Absolute Errors. All data were expressed as mean of three determinations.

The sorption constant, *K_L_*, and sorption capacity, *q_m_*, for Nd(III), Eu(III), La(III) are higher than those for Cs(I), Sr(II) and Tl(I), and this was interpreted as meaning that not all inorganic sites may be available for the last metal ions. For all studied metal ions, the maximum adsorption capacity is close to that experimental determined. This suggests that the Langmuir model provides a good model for the sorption of metal ions onto bone powder. The Langmuir adsorption model is established on the following hypotheses: uniformly energetic adsorption to bone powder, and no lateral interaction between adsorbed Nd(III), Cs(I), Sr(II), Tl(I), Eu(III) and La(III). The essential characteristic of the Langmuir isotherms can be expressed in terms of a dimensionless constant separation factor or equilibrium parameter, *R_L_*, which is defined as [18]:

(8) $$R_{L}=\frac{1}{\left(1+K_{L}C_{0}\right)}$$

*R_L_* value indicates the shape of the isotherm. *R_L_* values between 0 and 1 indicate favorable absorption [19], *R_L_* equal to 0 indicate irreversible absorption, *R_L_* = 1 is linear and *R_L_* > 1 is unfavorable. In this study, *R_L_* values for all studied metal ions adsorption are < 1, this indicates that the adsorption process of studied metal ions onto the bone powder is favorable.

The Freundlich equation frequently gives an adequate description of adsorption data over a restricted range of concentration, even though it is not based on any theoretical background. Apart from a homogeneous surface, the Freundlich equation is also suitable for a highly heterogeneous surface and an adsorption isotherm lacking a plateau, indicating a multi-layer adsorption [20].

Values of *1/n* less than unity are an indication that significant adsorption takes place at low concentration but the increase in the amount adsorbed with concentration becomes less significant at higher concentration and vice versa.

The magnitude of *K_F_* and *n*, shows that it is possible an easy separation of heavy metal ion from aqueous solution and a high adsorption capacity. For all studied metals the *1/n* is <1 suggesting the affinity of the bone powder for the studied metal ions.

Also, as the K_F_ value increases, so does the adsorption intensity.

Therefore, the higher *K_F_* values for Nd(III), Eu(III) and La(III) confirm by these models that the adsorption capacity of these is greater than that of the others ions. The results show that the Redlich-Peterson model agrees with the experimental data.

The values obtained for *β* are less than 1 for all adsorbate indicating an occurrence of a homogeneous predominated surface adsorption of the studied metal ions onto bone powder. In order to verify the model validity the *SSE*, *ARE* and *EABS* errors function were applied.

In case of the adsorption process of Nd(III), Eu(III), La(III) the ARE error function was found to be good for all the studied isotherms. For Cs(I), Sr(II) and Tl(I), adsorption process onto the SSE and EABS error function was found to be good for all the studied isotherm models. It can be observed that even for the adsorption process of Nd(III), Eu(III), La(III), higher correlation coefficients and higher adsorption capacity were obtained compared with the values obtained for Cs(I), Sr(II) and Tl(I) adsorption process. In these cases the values of the calculated errors are higher.

The Langmuir isotherm and Redlich–Peterson model were better than the Freundlich isotherm model. These models were able to describe perfectly experimental data.

Hence, it can be understood that, the Redlich-Peterson and Langmuir isotherms were the most suitable models for sorbate–sorbent system. A close correspondence was found between Langmuir and Redlich-Peterson isotherm models. Similar findings have also been reported by other researchers as well [21]. The experimental data of the adsorption equilibrium from Nd(III), Cs(I), Sr(II), Tl(I), Eu(III) and La(III) solution correlates with the using Langmuir isotherm equation, were supported by other authors [22]. The results for the Langmuir, Freundlich and Redlich-Peterson isotherm models were well contoured. The studies suggested that the metals’ adsorption onto bone powder was a spontaneous endothermic and physical reaction. Possibly, the economic aspect of the use of bone powder makes the re-use of these materials important when they are being re-used in the adsorption of metals [23,24].

## 4. Conclusions

The present investigation shows that bone powder is an effective adsorbent for the removal of studied metal ions from aqueous solutions. The equilibrium data have been analyzed using Langmuir, Freundlich and Redlich–Peterson isotherms. The characteristic parameters for each isotherm and related coefficients (*R^2^*) have been determined. The experimental data showed excellent fits within the following isotherms order: Langmuir > Redlich-Peterson > Freundlich, based on their coefficient of determination (*R^2^*) values. The bone powder developed higher adsorption performance in the removal process of Nd(III), Eu(III), La(III) from aqueous solutions than in the case of the removal process of Cs(I), Sr(II) and Tl(I) from aqueous solutions.
